# Cannabidiol Lipid Nanoparticles Stabilize Gut–Brain–Bone Axis Integrity and Enhance Neuroplasticity in Stressed Rats: A Comparison with Atomoxetine and Escitalopram

**DOI:** 10.3390/ijms26199318

**Published:** 2025-09-24

**Authors:** Sarawut Lapmanee, Jitpatima Lumsutti, Natthawut Charoenphon, Anjaree Inchan, Nittaya Boonmuen, Prapimpun Wongchitrat, Natchayaporn Thonapan, Chaowalit Yuajit, Piyaporn Surinlert, Chittipong Tipbunjong, Mattaka Khongkow, Katawut Namdee, Chaiyos Sirithanakorn

**Affiliations:** 1 Chulabhorn International College of Medicine, Thammasat University, Pathum Thani 12120, Thailand; piyaporn.latte@gmail.com; 2 Toxicology Graduate Program, Multidisciplinary Unit, Faculty of Science, Mahidol University, Bangkok 10400, Thailand; jitpatima.jl@gmail.com; 3 Department of Anatomy, Faculty of Medical Science, Naresuan University, Phitsanulok 65000, Thailand; natthawutch@nu.ac.th; 4 Faculty of Medicine, Praboromarajchanok Institute, Ministry of Public Health, Nonthaburi 11000, Thailand; anjaree.in@gmail.com; 5 Department of Physiology, Faculty of Science, Mahidol University, Bangkok 10400, Thailand; nittaya.bom@mahidol.ac.th; 6 Center for Research Innovation and Biomedical Informatics, Faculty of Medical Technology, Mahidol University, Nakhon Pathom 73170, Thailand; prapimpun.won@mahidol.ac.th; 7 Department of Radiological Technology, Faculty of Allied Health Sciences, Thammasat University, Pathum Thani 12120, Thailand; natchayaporn.t@allied.tu.ac.th; 8 College of Medicine and Public Health, Ubon Ratchathani University, Ubon Ratchathani 34190, Thailand; chaowalit.y@ubu.ac.th; 9 Thammasat University Research Unit in Synthesis and Applications of Graphene, Thammasat University, Pathum Thani 12120, Thailand; 10 Division of Health and Applied Sciences, Faculty of Science, Prince of Songkla University, Songkhla 90110, Thailand; chittipong.t@psu.ac.th; 11 National Nanotechnology Centre, National Science and Technology Development Agency, Pathum Thani 12120, Thailand; mattaka@nanotec.or.th; 12 Division of Molecular and Cellular Medicine, Faculty of Medicine, King Mongkut’s Institute of Technology Ladkrabang, Bangkok 10520, Thailand; chaiyos.si@kmitl.ac.th

**Keywords:** bone, cannabidiol lipid nanoparticles, gut metabolites, neuroinflammation, neuroplasticity, stressed rats

## Abstract

Chronic stress induces mood disturbances, disrupts gut barrier function, and promotes low-grade systemic inflammation. This study assessed the therapeutic effects of atomoxetine (ATX), escitalopram (ESC), cannabidiol (CBD), and CBD-loaded lipid nanoparticles (CBD/LNP) in male rats exposed to repeated restraint stress. Stressed rats exhibited a 2.03-fold increase in interleukin-6 and a 1.89-fold increase in TNF-α, a 1.20-fold decrease in brain-derived neurotrophic factor, a 1.36-fold decrease in osteocalcin, accompanied by alterations in gut metabolites, particularly short-chain fatty acids (SCFAs; from 155.3 to 94.83 μmol/L), polyamines (from 273.6 to 192.4 μmol/L), and bile acids (BAs; from 21.19 to 14.53 μmol/L), compared with the control group. Protein analysis revealed gut barrier disruption and microglial/macrophage activation, accompanied by reduced synaptic plasticity. ATX improved gut permeability and reduced glial activation but did not restore osteocalcin. ESC provided neuroimmune benefits with limited and BA gut restoration and modulated the gut–brain axis and improved anxiety-like behaviors, partly by altering gut microbiota and metabolites. CBD and CBD/LNP treatment restored intestinal barrier function, as indicated by intestinal permeability in the range of 1.15–1.61-fold. These treatments also normalized bile acids (1.0–1.38-fold) and osteocalcin (1.0–1.28-fold) and significantly reduced glial activation (0.63–1.12-fold) as opposed to the non-treated stressed group. All treatments were found to be effective in correcting SCFA and polyamine levels. Histological analysis confirmed that CBD/LNP, ATX, and ESC ameliorated tissue alterations. These findings highlight CBD/LNP as a promising intervention for stress-induced gut–brain–bone axis disruption, supporting its potential as a therapeutic alternative through modulation of microbiota-driven gut–brain communication in stress-associated disorders.

## 1. Introduction

Chronic physical and psychological stress exerts widespread effects on health, extending beyond neuropsychiatric manifestations to involve multiple physiological systems [1]. Prolonged hypothalamic–pituitary–adrenal (HPA) axis activation elevates glucocorticoids, impairing immunity, metabolism, neuroplasticity, gut barrier integrity, and bone remodeling [1,2,3,4]. These changes are framed within the gut–brain–bone axis, where dysfunction at one site propagates systemic pathology [4,5]. Stress disrupts gut integrity, enabling microbial translocation and inflammation [6], while dysbiosis alters short-chain fatty acids (SCFAs), polyamines, and bile acids (BAs) that control barrier function, neurogenesis, and host metabolism [7,8,9]. However, stress paradigms differ across models, leading to variable patterns of interorgan axis dysregulation.

Gut-derived inflammatory signals propagate systemically, activating gut-resident macrophages and brain microglia, as indicated by elevated Iba1/AIF-1 expression in stress-sensitive regions such as the hippocampus [10,11]. This neuroimmune activation is linked to deficits in cognition, emotional regulation, and synaptic plasticity, accompanied by reductions in glial cell line-derived neurotrophic factor and neurotrophic factors including brain-derived neurotrophic factor (BDNF) [3,12]. Chronic stress also impairs skeletal integrity by suppressing osteoblast activity and lowering circulating osteocalcin, a bone-derived hormone increasingly recognized for its regulatory roles in both neurocognitive and metabolic functions [13]. These neuroendocrine, immune, and microbial alterations often reinforce each other, creating a vicious cycle of systemic dysfunction.

Pharmacological interventions, especially selective serotonin reuptake inhibitors (SSRIs) and norepinephrine reuptake inhibitors (NRIs), are widely used to mitigate mood, attentional, and cognitive disturbances associated with chronic stress [14,15]. Escitalopram (ESC), a commonly prescribed SSRI, enhances neuroplasticity and emotional regulation [16] but may paradoxically exacerbate gut barrier dysfunction by increasing intestinal permeability, despite maintaining tight junction proteins such as zonula occludens-1 (ZO-1) and occludin [17]. Atomoxetine (ATX), an NRI less studied in gut contexts, improves central norepinephrine and dopamine balance, autonomic regulation, and gut metabolic function [18,19], potentially conferring additional benefits on bone remodeling and skeletal homeostasis under stress [20]. Bone integrity depends on balanced osteoblast and osteoclast activity regulated by systemic hormones and local turnover markers [21]. Modulation by SSRIs and NRIs via neurotransmission and neuroendocrine pathways has been reported to influence bone metabolism, although evidence remains inconsistent, with both protective and detrimental effects observed in stress-related models [22,23].

Beyond conventional antidepressants, phytocannabinoids, i.e., cannabidiol (CBD), a naturally occurring non-psychoactive phytochemical in *Cannabis sativa*, have gained increasing attention for their anti-inflammatory, anxiolytic, and neuroprotective properties in various neurological disorders [24]. CBD modulates glial activation, restores tight junction integrity, and upregulates BDNF, simultaneously addressing multiple aspects of chronic stress pathology [25,26,27]. Clinical application of CBD is limited by poor oral bioavailability, largely due to lipophilicity and extensive first-pass metabolism [28]. To overcome these limitations, lipid nanoparticle (LNP) formulations have been developed to enhance absorption, stability, and bioavailability [29,30]. Our recent findings indicate that CBD in LNPs improves metabolic and cognitive functions in rats with diabetic Parkinson’s disease, primarily through anti-inflammatory and neuroprotective mechanisms [29]; however, its potential in stress-induced models remains underexplored, particularly with respect to targeted delivery and sustained behavioral outcomes.

Although the individual effects of SSRIs, NRIs, and CBD on gut microbiota alterations have been reported [31,32,33], no study has systematically compared these interventions within a unified gut–brain–bone model. Additionally, the potential therapeutic advantage of CBD encapsulated in LNPs over its native form remains to be elucidated, particularly for restoring physiological and behavioral functions under chronic stress. The current study sought to evaluate repeated restraint stress on the gut–brain–bone axis and the therapeutic potential of ATX, ESC, and CBD administered in both native and LNP-encapsulated forms. We hypothesized that chronic stress would induce systemic inflammation, disrupt neuroimmune balance, impair synaptic plasticity, compromise gut integrity and bone remodeling, and cause histological alterations. We further expected that CBD/LNPs would reduce inflammation and provide superior therapeutic efficacy by restoring gut–brain barrier function, while improving behavioral, synaptic, neuroimmune, and skeletal outcomes, supporting CBD/LNPs as a multi-target strategy for stress-related comorbidities.

## 2. Results

### 2.1. Restraint-Stressed Male Rats Treated with CBD/LNPs Showed Modulated Body Weight Loss and Reduced Anxiety-like Behaviors, Comparable to the Effects Observed with Monoaminergic Modulators

**Figure 1:**
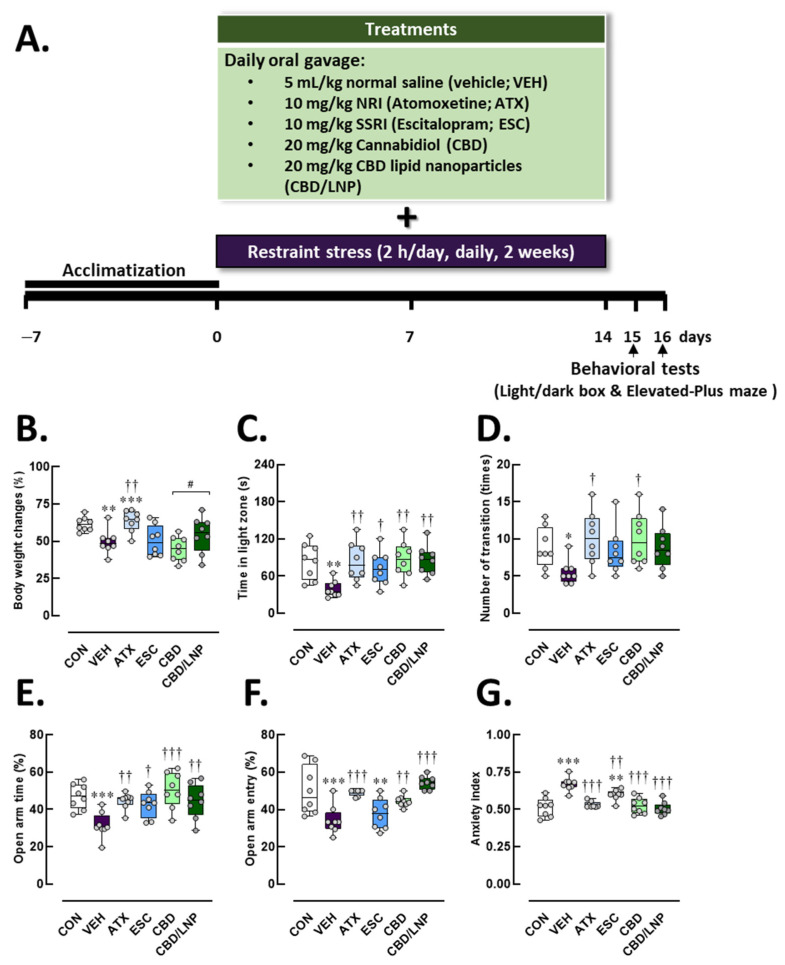
Physical and behavioral profiles after 14-day antipsychotic treatment in stressed male rats. (**A**) Diagrammatic representation of the experimental design. The experimental groups consisted of healthy control (CON), stress/vehicle (VEH, normal saline), stress/atomoxetine (ATX, 10 mg/kg), stress/escitalopram (ESC, 10 mg/kg), stress/cannabidiol (CBD, 20 mg/kg), stress/cannabidiol-loaded lipid nanoparticles (CBD/LNP, 20 mg/kg). (**B**) Percent body weight change. (**C**) Time spent in the light compartment and (**D**) number of transitions from the dark to light compartment in the light/dark box test. Open arms exploration (**E**) percentage of time spent in open arms and (**F**) percentage of entries. (**G**) Anxiety index in the elevated plus maze test. Results are presented as mean ± SEM (*n* = 8 per group). * *p* < 0.05, ** *p* < 0.01, *** *p* < 0.001 compared to CON group. † *p* < 0.05, †† *p* < 0.01, ††† *p* < 0.001 compared to VEH group, and # *p* < 0.05 compared to CDB group.

All rats maintained normal physical growth throughout the experimental period. However, after 14 days of 2 h daily restraint stress, significant changes were observed in both physical and behavioral parameters (Figure 1). Compared with controls, vehicle-treated stressed rats had a significantly lower percentage change in body weight (*p* < 0.01) (Figure 1B). Behavioral profiles demonstrated consistently elevated anxiety-like behaviors across test paradigms. The vehicle-treated stressed rats exhibited a marked reduction in time spent in the light compartment (*p* < 0.01) and fewer dark-to-light transitions in the light/dark box test (*p* < 0.05) (Figure 1C,D). Additionally, in the elevated plus maze (EPM) test, these rats spent a significantly lower percentage of time (*p* < 0.001) and made fewer entries (*p* < 0.001) into the open arms, accompanied by a markedly higher anxiety index (*p* < 0.001) (Figure 1E,F). In contrast, the 2-week antipsychotic intervention with ATX significantly improved body weight changes compared to the vehicle-treated stressed group (*p* < 0.01), whereas CBD/LNP treatment more effectively restored body weight in stressed rats compared to the natural form of CBD (*p* < 0.05). Compared to vehicle-treated stressed rats, all treatments produced anxiolytic-like effects, as demonstrated by behavioral profiles showing increased time spent in the light compartment (*p* < 0.01) and a higher number of transitions (*p* < 0.05) in the light/dark test, as well as increased time spent in the open arms (*p* < 0.001), increased open arm entries (*p* < 0.001), and a reduced anxiety index (*p* < 0.001) in the EPM test (Figure 1B–G). However, ESC might require a longer duration to effectively modulate central serotonergic signaling involved in anxiety attenuation, whereas adrenergic and endocannabinoid modulators exert more rapid and potent effects in stressed male rats.

### 2.2. Restraint-Stressed Male Rats Treated with CBD/LNPs Showed Improved Serum Biological Markers Related to Systemic Inflammation, Gut Permeability, Intestinal Metabolites, and Bone Remodeling, Comparable to the Effects Observed with Monoaminergic Modulators

**Figure 2:**
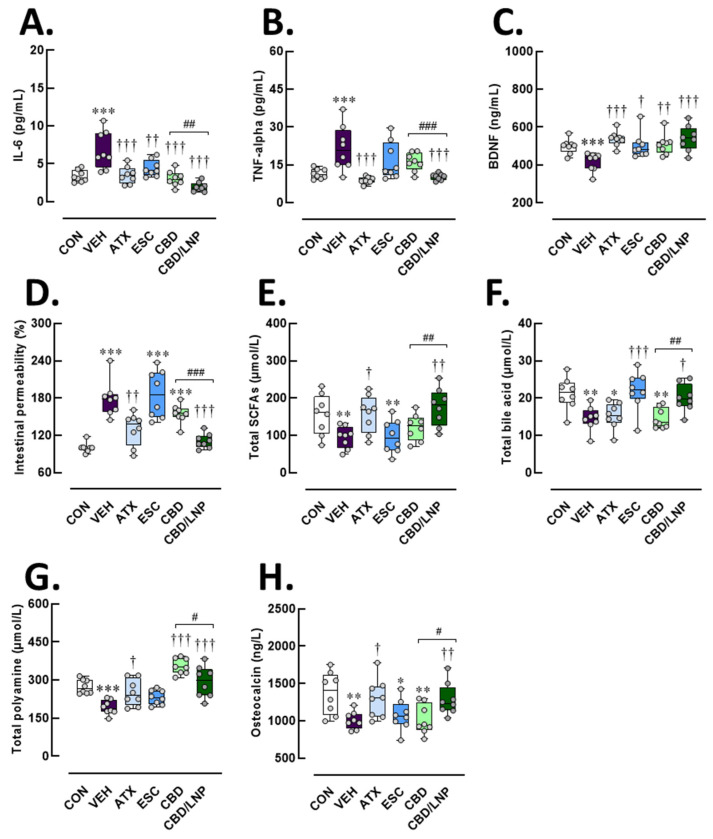
Serum biomarker profiles related to systemic inflammation, neuroimmune activity, neuroplasticity, intestinal function, gut-derived metabolites, and bone remodeling after 14-day antipsychotic treatment in stressed male rats. The experimental groups consisted of healthy control (CON), stress/vehicle (VEH, normal saline), stress/atomoxetine (ATX, 10 mg/kg), stress/escitalopram (ESC, 10 mg/kg), stress/cannabidiol (CBD, 20 mg/kg), stress/cannabidiol-loaded lipid nanoparticles (CBD/LNP, 20 mg/kg). (**A**) Interleukin-6 (IL-6), an inflammatory cytokine; (**B**) Tumor necrosis factor-alpha (TNF-α), a proinflammatory mediator, (**C**) Brain-derived neurotrophic factor (BDNF), (**D**) Relative intestinal permeability compared to control, (**E**) Short-chain fatty acids (SCFAs), (**F**) Total bile acids, (**G**) Total polyamines, and (**H**) Osteocalcin. Results are presented as mean ± SEM (*n* = 8 per group). * *p* < 0.05, ** *p* < 0.01, *** *p* < 0.001 compared to CON group. † *p* < 0.05, †† *p* < 0.01, ††† *p* < 0.001 compared to VEH group, # *p* < 0.05, ## *p* < 0.01 and ### *p* < 0.001 compared to CDB group.

In accordance with the anxiety-like behaviors observed in response to stress induction, these changes were associated with elevated serum levels of the proinflammatory cytokines IL-6 (*p* < 0.001) and TNF-α (*p* < 0.001) (Figure 2A,B), supporting the presence of systemic inflammation following chronic restraint. The vehicle-treated stressed rats exhibited significantly reduced BDNF levels (*p* < 0.001; Figure 2C), which were associated with anxiety-like behaviors, likely driven by neuroinflammation induced by chronic stress exposure. In relation to intestinal function, vehicle-treated stressed rats showed elevated serum fluorescein isothiocyanate (FITC)-dextran levels (*p* < 0.001; Figure 2D), indicating increased gut permeability. Moreover, stress induction disrupted intestinal metabolism, as reflected by significantly decreased levels of total SCFAs (*p* < 0.01), BAs (*p* < 0.001), and polyamines (*p* < 0.001) (Figure 2E–G). Additionally, stress negatively affected osteocalcin levels (*p* < 0.01; Figure 2H), a key hormone involved in bone remodeling, suggesting a potential interplay between central nervous system activity, behavioral responses, and bone metabolism. Following antipsychotic administration, all interventions reduced IL-6 levels (*p* < 0.001; Figure 2A); however, only ATX and CBD/LNPs significantly lowered TNF-α levels in stressed rats (*p* < 0.001; Figure 2B). Serum BDNF levels were restored in all treatment groups (*p* < 0.001; Figure 2C), suggesting a potential role in promoting neurogenesis in both central and peripheral organs after stress induction. In terms of intestinal outcomes, both ATX and CBD/LNPs effectively reduced gut permeability, as indicated by decreased serum FITC-dextran levels (*p* < 0.001), and restored levels of SCFAs (*p* < 0.01) (Figure 2D,E). Additionally, ESC and CBD/LNPs significantly increased serum BA levels (*p* < 0.001; Figure 2F), indicating improved gut metabolic activity. Restoration of polyamine levels was observed in the ATX and CBD treatment groups (i.e., CBD and CBD/LNPs) (*p* < 0.01), accompanied by increased osteocalcin levels, with the most pronounced effects seen in the ATX and CBD/LNP-treated rats (*p* < 0.01) (Figure 2G,H). Interestingly, CBD/LNPs exerted greater effects than CBD alone in reducing IL-6 (*p* < 0.01) and TNF-α (*p* < 0.001), as well as in improving polyamine levels (*p* < 0.05). Furthermore, CBD/LNPs demonstrated superior efficacy in restoring SCFAs (*p* < 0.01), BAs (*p* < 0.01), and osteocalcin (*p* < 0.05). These findings suggest that CBD/LNPs could offer broader systemic benefits, particularly in modulating the gut–brain–bone axis, similar to ATX and partially to ESC, with CBD demonstrating greater potency in anti-inflammatory responses.

### 2.3. Restraint-Stressed Male Rats Treated with CBD/LNPs Exhibited Improved Expression of Proteins Associated with Hippocampal and Colonic Barrier Integrity, Synaptic Plasticity, and Neuroimmune Inflammation, Comparable to the Effects Observed with Monoaminergic Modulators

**Figure 3:**
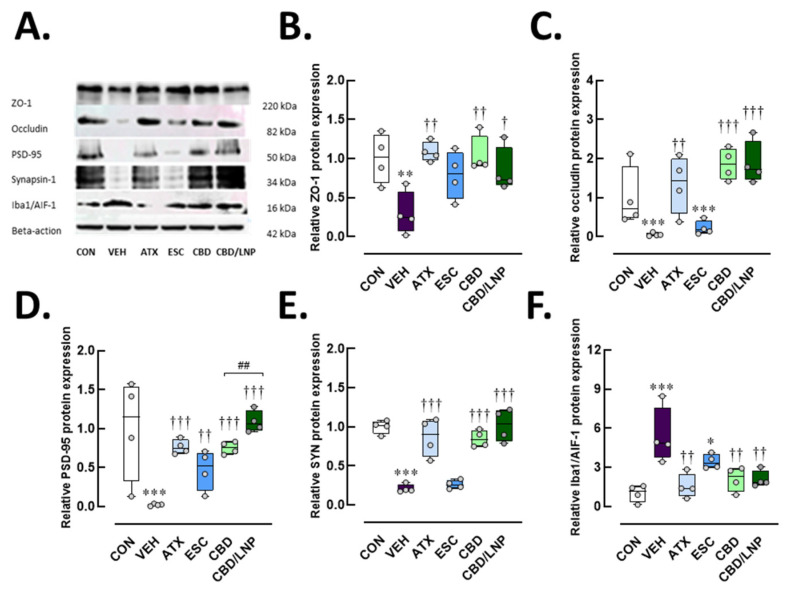
Protein expression profiles related to hippocampal integrity, synapsis, and neuroglial-mediated inflammation after 14-day antipsychotic treatment in stressed male rats. The experimental groups consisted of healthy control (CON), stress/vehicle (VEH, normal saline), stress/atomoxetine (ATX, 10 mg/kg), stress/escitalopram (ESC, 10 mg/kg), stress/cannabidiol (CBD, 20 mg/kg), stress/cannabidiol-loaded lipid nanoparticles (CBD/LNP, 20 mg/kg). (**A**) Representative Western blot images and respective quantification of (**B**) Zonula occludens-1 (ZO-1), a tight junction protein; (**C**) Occludin, a tight junction component; (**D**) Postsynaptic density protein 95 (PSD-95), a postsynaptic marker; (**E**) Synapsin-1 (SYN), a synaptic vesicle protein; (**F**) Ionized calcium-binding adapter molecule 1/Allograft inflammatory factor-1 (Iba1/AIF-1), a microglial marker. Results are presented as mean ± SEM (*n* = 4 per group). * *p* < 0.05, ** *p* < 0.01, *** *p* < 0.001 compared to CON group. † *p* < 0.05, †† *p* < 0.01, ††† *p* < 0.001 compared to VEH group, and ## *p* < 0.01 compared to CDB group.

Stressed rats treated with vehicle exhibited downregulation of hippocampal tight junction proteins, as shown in Figure 3A–C, including ZO-1 (*p* < 0.01) and occludin (*p* < 0.001). Reduced synaptic plasticity was also observed in the hippocampus (postsynaptic density protein 95, PSD-95, *p* < 0.001; Figure 3D, synapsin-1, *p* < 0.001; Figure 3E), accompanied by increased protein expression of the microglial activation marker Iba1/AIF-1 (*p* < 0.001; Figure 3F). As shown in Figure 3B–F, treatment with ATX effectively restored ZO-1 (*p* < 0.01), occludin (*p* < 0.001), PSD-95 (*p* < 0.001), synapsin-1 (*p* < 0.001), and Iba1/AIF-1 levels (*p* < 0.01). In contrast, ESC did not alter tight junction proteins, presynaptic markers, or microglia-mediated neuroinflammation. Both CBD and CBD/LNPs produced effects comparable to ATX, with CBD/LNPs showing greater efficacy than CBD alone, especially, in restoring PSD-95 postsynaptic neuronal markers (*p* < 0.01; Figure 3D). These results indicate that chronic stress compromises hippocampal barrier integrity and synapsis through neuroinflammatory mechanisms and suggest that CBD, particularly in its nanoparticle formulation, can offer enhanced neuroprotective potential in stress-induced hippocampal dysfunction.

**Figure 4:**
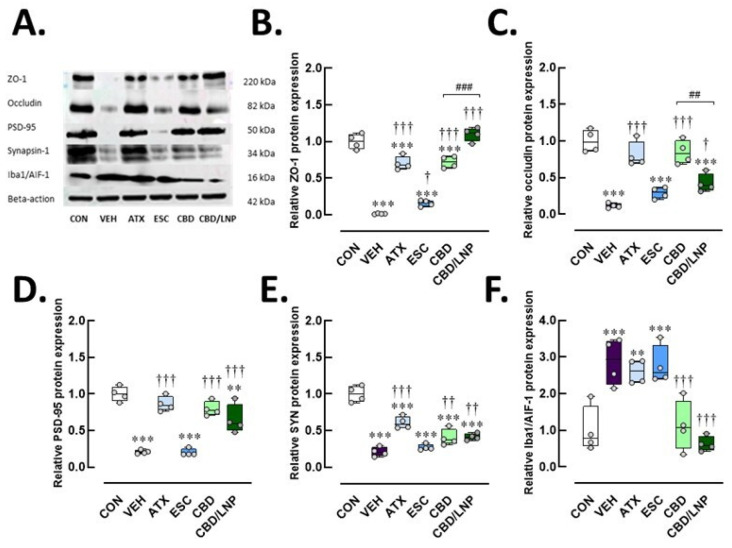
Protein expression profiles related to colonic integrity, synapsis, and neuroglial-mediated inflammation after 14-day antipsychotic treatment in stressed male rats. The experimental groups consisted of healthy control (CON), stress/vehicle (VEH, normal saline), stress/atomoxetine (ATX, 10 mg/kg), stress/escitalopram (ESC, 10 mg/kg), stress/cannabidiol (CBD, 20 mg/kg), stress/cannabidiol-loaded lipid nanoparticles (CBD/LNP, 20 mg/kg). (**A**) Representative Western blot images and respective quantification of (**B**) Zonula occludens-1 (ZO-1), a tight junction protein; (**C**) Occludin, a tight junction component; (**D**) Postsynaptic density protein 95 (PSD-95), a postsynaptic marker; (**E**) Synapsin-1 (SYN), a synaptic vesicle protein; (**F**) Ionized calcium-binding adapter molecule 1/Allograft inflammatory factor-1 (Iba1/AIF-1), a neuroimmune macrophage marker. Results are presented as mean ± SEM (*n* = 4 per group). ** *p* < 0.01, *** *p* < 0.001 compared to CON group. † *p* < 0.05, †† *p* < 0.01, ††† *p* < 0.001 compared to VEH group, and ## *p* < 0.01, ### *p* < 0.01 compared to CDB group.

In the colon (Figure 4A–E), the vehicle-treated stressed rats exhibited significantly downregulated expression of the tight junction protein ZO-1 (*p* < 0.001) and occludin (*p* < 0.001), and downregulation of synaptic plasticity markers PSD-95 (*p* < 0.001) and synapsin-1 (*p* < 0.001). These stressed rats also exhibited elevated Iba1/AIF-1 macrophage-associated inflammation in colonic tissues (*p* < 0.001; Figure 4F). Among treatments, ATX—but not ESC—significantly restored the expression of ZO-1 (*p* < 0.001), occludin (*p* < 0.001), PSD-95 (*p* < 0.001), and synapsin-1 (*p* < 0.001) compared to the vehicle group (Figure 4B–E). Similar restorative effects were observed with CBD treatment, especially with CBD/LNPs, demonstrating the most pronounced enhancement of ZO-1 (*p* < 0.001), while the natural form of CBD more effectively improved occludin expression (*p* < 0.01) (Figure 4B,C). Notably, ATX and ESC had no significant effect on Iba1/AIF-1 levels, whereas both CBD and CBD/LNPs significantly reduced Iba1/AIF-1 protein levels (*p* < 0.001; Figure 4F), indicating attenuation of stress-induced macrophage activation. These findings suggest that CBD, particularly in its LNP form, offers greater therapeutic potential for restoring intestinal barrier integrity and synaptic plasticity while effectively reducing gut-associated neuroinflammation under chronic stress conditions.

### 2.4. Restraint-Stressed Male Rats Treated with CBD/LNPs Exhibited Improved Histomorphological Changes in the Hippocampus, Colon, and Tibia, Comparable to the Effects Observed with Monoaminergic Modulators

**Figure 5:**
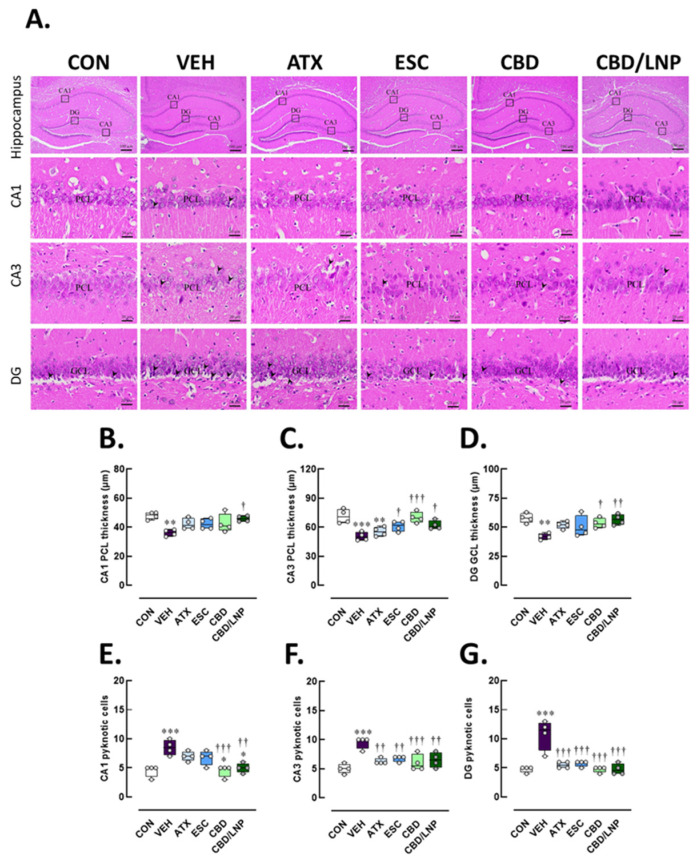
Histomorphological consequences of hippocampal tissues after 14-day antipsychotic treatment in stressed male rats. The experimental groups consisted of healthy control (CON), stress/vehicle (VEH, normal saline), stress/atomoxetine (ATX, 10 mg/kg), stress/escitalopram (ESC, 10 mg/kg), stress/cannabidiol (CBD, 20 mg/kg), stress/cannabidiol-loaded lipid nanoparticles (CBD/LNP, 20 mg/kg). (**A**) Representative histological images of the hippocampus processed for hematoxylin and eosin (H&E) staining. (**B**) Thickness of the pyramidal cell layer (PCL) in the cornu ammonis 1 (CA1) region, (**C**) thickness of the PCL in the CA2 region, (**D**) thickness of the granule cell layer (GCL) in the dentate gyrus (DG). (**E**) Number of pyknotic cells in the CA1 region, (**F**) number of pyknotic cells in the CA2 region, and (**G**) number of pyknotic cells in the DG region of the hippocampus. Results are presented as mean ± SEM (*n* = 4 per group). * *p* < 0.05, ** *p* < 0.01, *** *p* < 0.001 compared to CON group. † *p* < 0.05, †† *p* < 0.01, ††† *p* < 0.001 compared to VEH group. Arrowhead (⮞) indicates the pyknotic cells. The scale bar represents 50 µm at 400× magnification.

Histological analysis of hippocampal sections revealed distinct histopathological changes across the cornu ammonis 1 (CA1), cornu ammonis 3 (CA3), and dentate gyrus (DG) regions of the hippocampus among experimental groups (Figure 5A). In the control group, the hippocampal cytoarchitecture was well preserved. The pyramidal cell layer (PCL) in both CA1 and CA3 regions appeared densely packed and orderly, with uniform cell morphology and no evidence of nuclear condensation. Similarly, the GCL in the DG exhibited tightly arranged granule neurons with minimal intercellular space and the absence of pyknotic cells. In contrast, the vehicle-treated stressed group displayed pronounced neuronal injury. The PCL in CA1 and CA3 appeared disrupted and loosely arranged, with lower PCL thickness (CA1, *p* < 0.01; CA3, *p* < 0.001) (Figure 5B,C), and also a notable presence of pyknotic nuclei, indicative of neuronal degeneration (CA1, *p* < 0.001; CA3, *p* < 0.001) (Figure 5E,F). The GCL of the DG also showed signs of disorganization, with reduced granule cell thickness (*p* < 0.01; Figure 5D) and a higher number of pyknotic cells as compared with controls (*p* < 0.001; Figure 5G). Treatment with ATX led to a partial restoration of hippocampal structure (Figure 5B–C). The PCL thickness in CA3 was greater and more compactly aligned in the ATX-treated stressed group relative to the vehicle group, although occasional pyknotic cells were still observed in CA3 (*p* < 0.01) (Figure 5C,F). In addition, the granule cell layer (GCL) of the DG exhibited improved granule cell organization (Figure 5D) accompanied by a reduction in pyknotic cells (*p* < 0.001; Figure 5F). Similarly, ESC treatment improved neuronal morphology, especially in CA3 hippocampal subregions. The PCL in CA3 appeared more intact (*p* < 0.001) with fewer pyknotic cells in the ESC-treated stressed group relative to the vehicle-treated stressed group (*p* < 0.01, Figure 5C,F), while the DG exhibited restored pyknotic cells (*p* < 0.001, Figure 5E). Treatment with CBD (i.e., CBD and CBD/LNPs) further improved histological features. The CA1 and CA3 regions showed enhanced cellular alignment with restored PCL thickness (CBD/LNPs, CA1, *p* < 0.05; CBD, *p* < 0.001, and CBD/LNPs, *p* < 0.05, CA3) (Figure 5B,C), and GCL of the DG appeared more densely packed (CBD, *p* < 0.05, CBD/NLPs, *p* < 0.01, Figure 5D). Both CBD and CBD/LNPs reduced the incidence of pyknotic nuclei in CA1, CA3 and DG (*p* < 0.001; Figure 5E–G). Notably, CBD—particularly when delivered via lipid-based nanocarriers—exhibited the most pronounced neuroprotective effects, as demonstrated by highly organized neuronal layers of the hippocampus and well-preserved cellular integrity, comparable to those observed with ATX and ESC.

**Figure 6:**
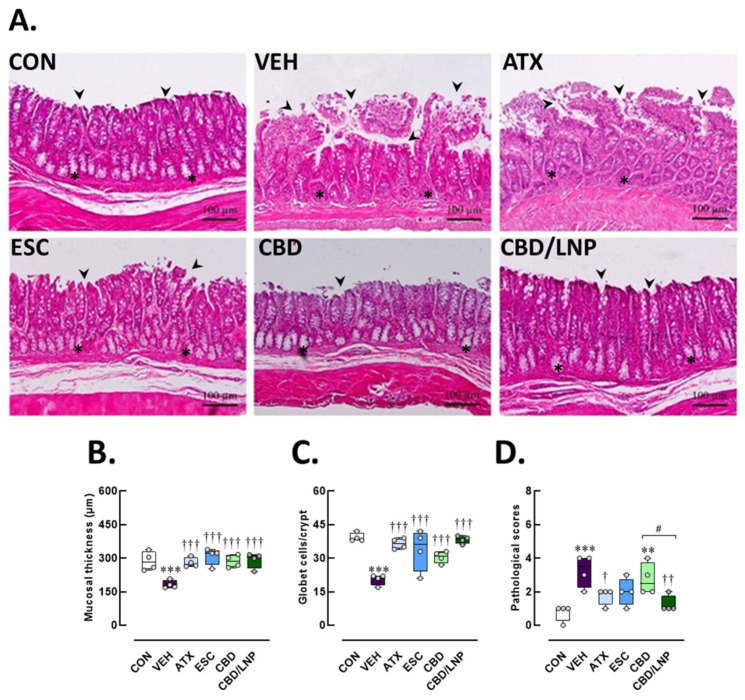
Histomorphological consequences of colon tissues after 14-day antipsychotic treatment in stressed male rats. The experimental groups consisted of healthy control (CON), stress/vehicle (VEH, normal saline), stress/atomoxetine (ATX, 10 mg/kg), stress/escitalopram (ESC, 10 mg/kg), stress/cannabidiol (CBD, 20 mg/kg), stress/cannabidiol-loaded lipid nanoparticles (CBD/LNP, 20 mg/kg). (**A**) Representative histological images of the colon tissue processed for hematoxylin and eosin (H&E) staining. (**B**) Thickness of the mucosal layer, (**C**) goblet cell count per crypt, and (**D**) total histopathological score. Results are presented as mean ± SEM (*n* = 4 per group). ** *p* < 0.01, *** *p* < 0.001 compared to CON group. † *p* < 0.05, †† *p* < 0.01, ††† *p* < 0.001 compared to VEH group and # *p* < 0.01 compared to CDB group. Arrowhead (⮞) indicates epithelium lining and asterisks (*) indicates the crypts in colonic tissues. The scale bar represents 100 µm at 400× magnification.

As shown in Figure 6A, the colonic mucosa maintained its normal histoarchitecture in the control group. The crypts were well-organized, extending vertically from the muscularis mucosae to the luminal surface. The epithelial lining remained intact, and goblet cells were abundantly and uniformly distributed along the crypts, reflecting optimal mucosal function. The submucosa appeared normal, with no signs of inflammation, crypt distortion, or epithelial damage. In contrast, the vehicle-treated stressed group displayed prominent stress-induced pathological alterations. Significant mucosal damage was observed in the vehicle-treated stressed group, reflected by thinner mucosa (*p* < 0.001; Figure 6B) and fewer goblet cells (*p* < 0.001; Figure 6C). Inflammatory cell infiltration was extensively observed in the vehicle-treated group, resulting in an increase in pathological scores (*p* < 0.001; Figure 6D).

All treatments markedly restored mucosal thickness (*p* < 0.001) and goblet cell numbers (*p* < 0.001) compared to the vehicle group, as shown in Figure 6B,C. ATX provided potent protection against these changes, as demonstrated by improved mucosal architecture (*p* < 0.001) and preserved goblet cell count (*p* < 0.001) relative to the non-treated stressed group. Comparable improvements in pathological scores were also observed with ATX (*p* < 0.05; Figure 6D), indicating a moderate therapeutic benefit. Although ESC treatment resulted in greater histological preservation, including mucosal thickness (*p* < 0.001) and goblet cell count (*p* < 0.001), it provided limited protection against inflammatory infiltration, as reflected by minimal changes in pathological scores (Figure 6B–D). These findings suggest that ESC could help ameliorate stress-induced colonic injury; however, a longer treatment duration might be necessary to achieve optimal efficacy. Remarkably, the group treated with natural-form CBD demonstrated substantial improvement in mucosal colonic tissue structure (*p* < 0.001). The crypts remained mostly intact, and goblet cell density approached levels (*p* < 0.001). Furthermore, inflammatory infiltration in the CBD-treated group was minimal, and the lamina propria appeared less expanded, supporting the notion that CBD exerts a specific, targeted protective effect on the colonic mucosa (Figure 6A–D).

Lastly, a potent restorative effect appeared in the CBD/LNP-treated stressed group, with colonic architecture nearly fully restored to resemble that of the control group. The epithelial surface remained continuous and intact as mucosal thickness was restored (*p* < 0.001). Crypts appeared well-formed, regularly aligned, and densely populated with goblet cells (*p* < 0.001). These effects were associated with significantly lower pathological scores (*p* < 0.01; Figure 6D). Therefore, CBD/LNPs provided the most effective protection, likely due to enhanced mucosal delivery. Taken together, the results confirmed that vehicle-treated stressed male rats exhibited significant tissue damage, validating the stress-induced colonic injury model. ATX and ESC conferred moderate therapeutic benefits, while both natural CBD and CBD/LNPs provided substantial histological preservation. Notably, CBD/LNPs offered greater mucosal protection than CBD alone (Figure 6D), emphasizing the potential advantage of nanoparticle-based delivery systems in mitigating colonic inflammation and stress-induced mucosal and goblet cell injury.

**Figure 7:**
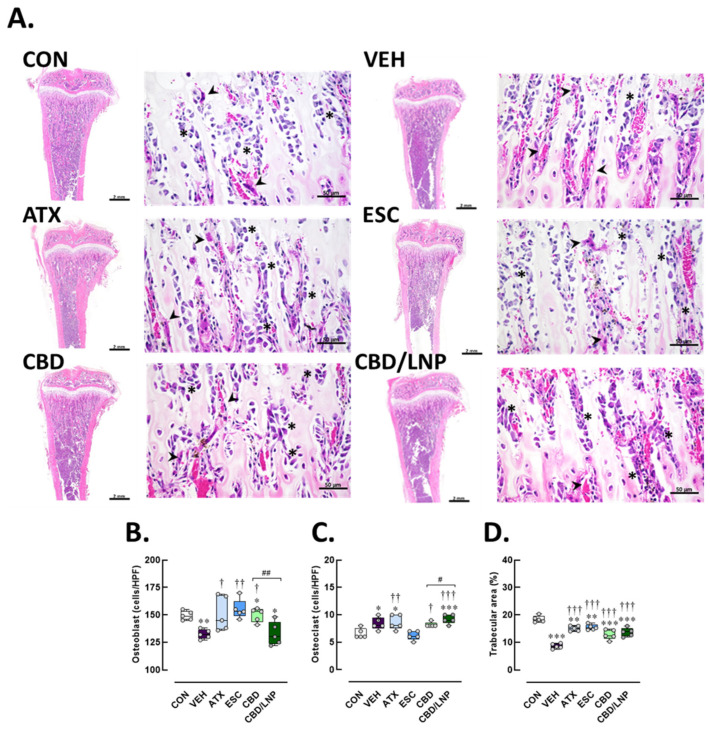
Histomorphological consequences of tibial bone tissues after 14-day antipsychotic treatment in stressed male rats. The experimental groups consisted of healthy control (CON), stress/vehicle (VEH, normal saline), stress/atomoxetine (ATX, 10 mg/kg), stress/escitalopram (ESC, 10 mg/kg), stress/cannabidiol (CBD, 20 mg/kg), stress/cannabidiol-loaded lipid nanoparticles (CBD/LNP, 20 mg/kg). (**A**) Representative histological images of tibia processed for hematoxylin and eosin (H&E) staining. (**B**) Number of osteoblasts, (**C**) number of osteoclasts, and (**D**) percentage of trabecular bone area. Results are presented as mean ± SEM (n = *4* per group). * *p* < 0.05, ** *p* < 0.01, *** *p* < 0.001 compared to CON group. † *p* < 0.05, †† *p* < 0.01, ††† *p* < 0.001 compared to VEH group, # *p* < 0.05 and ## *p* < 0.01 compared to CDB group. Arrowhead (⮞) indicates the osteoclasts and asterisks (*) indicates the osteoblasts. The scale bar represents 50 µm or 2 mm at 400× magnification.

Histological examination of longitudinal tibial sections revealed significant alterations in bone microarchitecture and cellular composition across the experimental groups (Figure 7). In the control group, the trabecular bone appeared thick and well-organized, forming an interconnected network beneath the growth plate. Numerous osteoblasts, identifiable as basophilic, cuboidal cells, were lined along the trabecular surfaces, indicating active bone formation. Osteoclasts were present in low numbers, appearing as large multinucleated cells situated in resorption lacunae (Figure 7A). Conversely, the vehicle-treated group exhibited a notable decrease in the number of osteoblasts, which appeared flattened and disorganized (*p* < 0.01; Figure 7B). In parallel, there was an increase in osteoclast numbers (*p* < 0.05; Figure 7C), characterized by large multinucleated cells actively resorbing bone, suggesting enhanced bone turnover and resorption. A marked decrease in trabecular area accompanied these alterations (*p* < 0.001; Figure 7D), indicating an increased risk of bone loss associated with chronic stress-induced inflammation.

As shown in Figure 7A–C, treatment with ATX partially restored bone microstructural architecture accompanied by a significant rise in osteoblast count (*p* < 0.05), while osteoclast density remained slightly elevated (*p* < 0.01), suggesting a shift toward bone regeneration. In Figure 7D, trabecular bone structure was better preserved in ATX-treated stressed rats compared with vehicle-treated stressed rats (*p* < 0.001). These findings suggest that ATX exerts a bone-protective effect under stress-induced conditions. The ESC-treated group, however, showed marked improvement, characterized by increased osteoblast presence (*p* < 0.01) and a tendency toward reduced osteoclast numbers, reflecting a shift toward balanced bone remodeling (Figure 7B,C). Trabecular areas were more effectively preserved and expanded following ESC treatment in stressed rats compared with vehicle-treated rats (*p* < 0.001; Figure 7D). Notably, treatment with CBD significantly increased osteoblast numbers (*p* < 0.05; Figure 7B) and decreased osteoclast numbers (*p* < 0.05; Figure 7B), contributing to improved trabecular morphology. As shown in Figure 7D, the trabecular bone area was markedly restored (*p* < 0.001), supporting the bone-protective effects of CBD under stress-induced conditions. Although osteoblast numbers were not significantly altered in the CBD/LNP-treated group, osteoclast numbers were significantly increased (*p* < 0.001; Figure 7B,C). These results suggest enhanced bone turnover and accelerated remodeling, as indicated by an increase in trabecular area (*p* < 0.001; Figure 7D).

## 3. Discussion

Chronic physical and psychological stress is known to provoke anxiety, depression, and cognitive impairments in both rodents and humans through stimulation of the hypothalamic–pituitary–adrenal (HPA) axis and an increase in systemic glucocorticoids [1,2,3]. This neuroendocrine imbalance has widespread effects on brain function and systemic homeostasis. In this study, a further ongoing investigation involving 14 days of repeated restraint stress combined with short-acting antipsychotic administration in male rats led to a triad of physiological impairments across the gut–brain–bone axis, mirroring clinical manifestations of chronic stress-related disorders.

Stressed rats showed significant reductions in body weight gain, demonstrated by robust anxiety-like behaviors and diminished exploratory activity in both the light/dark box and the elevated-plus maze tests (EPM) (Figure 1B–G), which is consistent with previous reports of learned fear, depressive-like behavior, and impaired learning and memory [3,34]. These mixed behavioral phenotypes validate the successful establishment of chronic stress-induced anxiety through the applied stress protocol. Importantly, neuroanatomical and molecular analyses revealed substantial stress-related neuropathology. Stressed male rats exhibited reduced dendritic spine density, particularly of mushroom spines, in the amygdala [35], alongside elevated levels of corticosterone, norepinephrine, and inflammatory markers, including IL-6 and TNF-α (Figure 2A,B), as well as C-reactive protein and stromal cell-derived factor-1, confirming systemic immune activation [36,37].

Neuroplasticity was compromised, as shown by decreased serum brain-derived neurotrophic factor (BDNF) levels (Figure 2C), which are closely linked with mood and cognitive regulation [38,39,40]. The hippocampus displayed hallmark features of neuroinflammation and degeneration, including reduced hippocampal thickness, downregulated synaptic (PSD-95 and synapsin-1) and tight junction proteins (ZO-1 and occludin), and increased expression of Iba1/AIF-1, a marker for microglial/macrophage activation (Figure 3 and Figure 5). These changes suggest that chronic stress disrupts both blood–brain barrier integrity and synaptic connectivity, contributing to behavioral and cognitive deterioration.

Furthermore, the differential expression of neuroimmune Iba1/AIF-1 observed between hippocampal and colonic tissues, particularly the persistent upregulation of this protein in the atomoxetine (ATX)- and escitalopram (ESC)-treated groups, could reflect tissue-specific neuroimmune dynamics. This pattern is likely attributable to the heightened sensitivity of the gastrointestinal tract to stress-induced immune activation (Figure 4F), which promotes enhanced infiltration of peripheral immune cells and activation of enteric glial cells. In contrast, microglial activation within the brain, especially in the hippocampus, typically shows a delayed or attenuated response because of the modulatory effects of the neurovascular barrier and region-specific signaling mechanisms. Consequently, the elevated Iba1 expression remains evident in the treatment groups [41]. It is also plausible that classical ESC exerts the therapeutic effects through gradually developing neuroadaptive processes, necessitating a longer duration to achieve sustained immunomodulatory outcomes compared to more rapidly acting agents, i.e., cannabidiol (CBD) and cannabidiol-loaded lipid nanoparticles (CBD/LNPs).

Beyond the brain, our findings reveal stress-induced disruption of gut homeostasis. Consistent with previous studies [3,42], stress reduced tight junction protein expression, compromising intestinal barrier integrity. This was reflected by mucosal damage, goblet cell loss, increased serum fluorescein isothiocyanate (FITC)-dextran, shifts in gut microbiota, and decreased levels of key microbiota-derived metabolites, i.e., short-chain fatty acids (SCFAs), polyamines, and bile acids (BAs) (Figure 2D–G and Figure 6).

Gut dysbiosis in depression also disrupts the microbial metabolome. BA levels have been linked to more severe depressive symptoms and cancer [9,43], while levels of SCFAs (e.g., acetate, propionate, and butyrate) are often reduced [44,45]. Additionally, physiological stressors like aging can lead to a decline in polyamine levels, further compromising gut and systemic health. However, supplementation or modulation of gut microbiota can restore polyamine levels, thereby enhancing stress resilience across multiple organs [46,47]. Notably, butyrate has demonstrated antidepressant-like effects in rodents by improving intestinal barrier integrity and reducing stress responsiveness. Furthermore, probiotic or synbiotic interventions (e.g., *Lactobacillus farciminis*) have been shown to prevent gut permeability and attenuate HPA axis activity [48].

Collectively, the findings indicate that stress-related intestinal barrier impairment facilitates systemic and neuroinflammation through gut–brain communication, potentially exacerbated by gut microbial dysbiosis. Concomitant with gut–brain disruption, the bone compartment also suffered. Rats exposed to restraint stress displayed diminished osteoblast activity, elevated osteoclast numbers, and reduced trabecular bone area (Figure 7). Elevated IL-6 and TNF-α levels likely interfered with bone cell signaling, suppressing bone formation while promoting resorption [49]. These skeletal alterations are consistent with previous reports showing that glucocorticoid excess and low-grade inflammation impair bone remodeling by elevating circulating parathyroid hormone levels and activating phospholipase C and the proinflammatory nuclear factor kappa-light-chain-enhancer of activated B cell pathways [37,50,51].

Pharmacologically, conventional monoaminergic agents, i.e., ATX and ESC demonstrated efficacy in reversing several stress-induced impairments. ATX improved weight loss, while both drugs alleviated anxiety behaviors (Figure 1B,C) and elevated BDNF levels (Figure 2C), consistent with their established roles in modulating adrenergic and serotonergic signaling [20,52,53]. However, ESC had delayed effects on gut and neuroinflammation, possibly reflecting the time required for serotonergic adaptation [16].

In contrast, CBD, particularly in its lipid nanoparticle form, CBD/LNPs, exhibited rapid and robust therapeutic effects. CBD is known for its inflammation-reducing, antioxidant, and neuroprotective activities [54], and previous studies support its efficacy in attenuating neuroinflammation in models of stress and neurodegeneration [55,56]. Our results showed that CBD/LNPs outperformed native CBD in restoring body weight, attenuating anxiety-like behavior, improving intestinal permeability, and enhancing hippocampal morphology (Figure 1, Figure 2, Figure 3, Figure 4 and Figure 5). These findings suggest enhanced bioavailability and targeted delivery conferred by the nanoformulation [29,57].

Either CBD or CBD/LNPs restored SCFA and BA levels while reducing serum FITC-dextran concentrations, thereby reestablishing gut barrier integrity and tight junction function [26]. However, CBD was more effective in reducing proinflammatory cytokines IL-6 and TNF-α, demonstrating anti-inflammatory activity, while CBD/LNPs excelled in restoring metabolic markers such as polyamines and osteocalcin [33,58]. This dual efficacy suggests that native CBD primarily acts through CB1/CB2 and TRPV1 receptors [59], while LNP-based delivery enhances systemic impact via improved bioavailability and tissue penetration.

Therapeutic modulation of skeletal outcomes was observed in this study. ATX partially improved trabecular deterioration in the tibiae via noradrenergic stimulation of osteoblasts, whereas ESC provided more robust protection by reducing osteoclastogenesis and preserving trabecular microarchitecture through serotonin-dependent pathways [20,22]. Notably, CBD and CBD/LNPs markedly preserved bone density; CBD enhanced osteoblast activity and reduced resorption, while CBD/LNPs further improved outcomes by mitigating inflammation and targeting local bone environments, consistent with nanoparticle-based bone regeneration strategies [60,61,62]. These findings demonstrate drug-specific modulation of neuroendocrine–bone crosstalk under stress.

As aforementioned, the enhanced effects of CBD/LNP compared to native CBD were more evident in peripheral tissues, consistent with reports that lipid-stabilized nanoparticles improve efficacy in an imiquimod-induced psoriasis model [63] and enhance intestinal bioaccessibility [64]. This likely reflects the pharmacological properties of LNPs, which increase systemic bioavailability and tissue distribution, whereas the capacity to penetrate the blood–brain barrier may be more limited. Although unloaded LNPs were not tested, prior studies indicate minimal biological effects [29,30,65], supporting that the observed benefits are primarily due to encapsulated CBD. Overall, these findings suggest that CBD/LNPs provide multi-organ protection with faster-acting, broader systemic efficacy compared to conventional drugs.

Although SSRIs, NRIs, and CBD act on different primary targets, these agents may converge on common downstream pathways that restore stress-induced impairments, with CBD sometimes showing superior efficacy due to its multi-target properties. Synergistic interactions are also plausible, as co-treatment with CBD and fluoxetine, desipramine, or ketamine has been shown to enhance antidepressant effects in mice [66,67], and we plan to investigate such combinations in future studies.

Nonetheless, this study has several limitations. Only male rats were used, as the estrous cycle in females can influence behavioral responses to interventions [3,68], introducing variability and precluding evaluation of sex-specific effects. Long-term outcomes, such as symptom relapse or development of treatment tolerance, were not assessed, and key molecular mechanisms—including apoptosis, neurogenesis, and gut microbiota profiling—remain unexplored. Although the chronic restraint stress model effectively induces anxiety-like behaviors, this model might not fully reflect the complexity of human stress-related psychiatric disorders. Further studies should address these gaps by employing longitudinal, multi-modal approaches in both male and female subjects.

## 4. Materials and Methods

### 4.1. Cannabidiol-Loaded Lipid Nanoparticles (CBD/LNPs) Preparation

CBD was purchased from Salus Bioceutical (Thailand) Co., Ltd. Bangkok, Thailand. CBD/LNPs were prepared using a solvent injection method following a previous study [29]. In brief, CBD isolate (Lot no. A24073) was analyzed and found to contain 99.64% *w*/*w* total CBD using an in-house HPLC method followed the Association of Official Agricultural Chemists (AOAC) official method at the Center for Analytical Testing of Medical Cannabis and Narcotic Plants, Thailand. CBD was dissolved in ethanol with lipid components—phosphatidylcholine (Lipoid GmbH, Ludwigshafen, Germany) and cholesterol. The organic phase was mixed with an aqueous phase (deionized water) using a high-speed homogenizer (IKA, Staufen, Germany), followed by size reduction through microfluidization (M-110P Microfluidizer, Microfluidics Inc., Westwood, MA, USA). LNPs were formed mechanically, and ethanol was removed by rotary evaporation under reduced pressure, producing a CBD concentration of 3 mg/mL. Encapsulation efficiency of CBD/LNP was determined using a membrane filter and centrifugation. The supernatant containing unencapsulated CBD was removed, and encapsulated CBD was quantified by HPLC-UV. Transmission electron microscopy was used to examine morphology, and particle size, polydispersity index (PDI), and zeta potential were measured via dynamic light scattering (DLS) using a Malvern Zetasizer Nano ZX (Malvern, UK). Particles exhibited a size ≤ 170 nm, PDI ≤ 0.2, and a zeta potential of −16.57 ± 0.04 mV. CBD-loaded lipid nanoparticles (CBD/LNP) remained stable for 1 month, with an encapsulation efficiency of 98.78 ± 0.90%, consistent with CBD’s high hydrophobicity [29].

### 4.2. Animal

Since rats are widely used as animal models of stress to mimic human responses, forty-eight adult sexually mature male Wistar rats (8 weeks old, weighing 200–210 g; Nomura Siam International Company Limited, Bangkok, Thailand) were used. Rats were kept three per polycarbonate cage with stainless-steel tops to minimize stress and fear. The rats were housed under a 12/12 h light (245 ± 5 lux)/dark cycle at 24 ± 1 °C and 54 ± 5% humidity. Rats were given unrestricted access to a standard rodent food (CP Company Limited, Bangkok, Thailand) and water. All animal procedures were approved by the Animal Care and Use Committee of Thammasat University, Pathumthani, Thailand (Animal Ethics number 003/2020, renewed in 2024).

### 4.3. Experimental Design

After 7 days of acclimatization, the rats were distributed randomly into six experimental groups, each containing eight animals (*n* = 8), a number determined by statistical power analysis to meet the minimum required sample size: (*i*) non-stressed control group (CON), (*ii*) stressed + vehicle group (VEH), (*iii*) stressed + atomoxetine group (ATX), (*iv*) stressed + escitalopram group (ESC), (*v*) stressed + CBD group (CBD), and (*vi*) stressed + CBD lipid nanoparticles group (CBD/LNPs). Animals were immobilized in a restrainer for 2 h/day, coupled with oral administration of antipsychotic agents for 14 days [3,20]. Careful monitoring was conducted in accordance with the refinement principle of the 3Rs to minimize suffering throughout the study. Any animals exhibiting signs of illness, reduced food or water intake, significant body weight loss, or impaired mobility would have been excluded; however, no exclusions were necessary, as no adverse effects were observed. An overview of the experimental workflow is presented in Figure 1A.

The final doses of antipsychotic regimens were administered 24 h prior to behavioral testing. Twenty-four hours post-behavioral testing, rats were fasted for at least 6 h and orally administered fluorescein isothiocyanate (FITC)-dextran to assess intestinal permeability, followed by blood collection via cardiac puncture for biochemical analyses. Behavioral assessments were carried out in the morning hours, from 9:00 a.m. to 12:00 p.m., and treatments were administered in the afternoon (3:00–4:00 p.m.) to minimize acute stress and accurately assess therapeutic efficacy.

Subsequently, rats were euthanized under isoflurane anesthesia. The hippocampus was collected for synaptic, tight junction, and neuroimmune protein analyses (*n* = 4/group), while an additional four hippocampal samples were perfused via cardiac injection with a fixative solution. for histological and neuronal integrity assessment. Additionally, two 1 cm colon sections were collected for histology and protein analyses, and the right tibiae were harvested for histological evaluation.

### 4.4. Stress Induction

Each rat underwent immobilization in a 24 × 6 cm transparent plastic cylinder, secured with bright plastic tape, for 2 h per day over 2 weeks [20,34,69]. Each cylinder had an end hole of 1 cm diameter for respiration. Restraint sessions were conducted daily from 9:00 to 11:00 a.m. in a controlled environment to minimize additional stress and were performed separately from the control group. Control animals were handled using familiar procedures similar to those applied to the stress group. Restraint was applied prior to antipsychotic administration to induce anxiety-like behaviors.

### 4.5. Antipsychotic Administration

The anti-stress drugs, atomoxetine (ATX) and escitalopram (ESC), were purchased from Lilly Del Caribe Inc., Carolina, Puerto Rico, and Sun Pharmaceutical Industries Ltd., Mumbai, India. Based on previous protocol by Songphaeng et al. [20], ATX and ESC have been shown to mitigate depression-like behavior in the inescapable forced swimming test. For this study, all treatment solutions were freshly prepared using sterile normal saline as the solvent, which also served as the vehicle control. Rats in the control (CON) and stressed vehicle (VEH) groups received 5 mL/kg normal saline by oral gavage. Stressed rats in the treatment groups received 10 mg/kg of either ATX or ESC. The CBD isolated powder and CBD/LNPs treatments were given by oral gavage at 20 mg/kg/day, representing the precise amount of CBD encapsulated in the lipid nanoparticles. Considering the encapsulation efficiency of the CBD/LNPs at 98.78 ± 0.90% [29], the total amount of CBD/LNP administered was calculated to be approximately 7 mg, delivered in a volume of 2.36 mL per rat weighing 350 g. To compare the therapeutic efficacy among these regimens, treatments were administered after the stress period to avoid acute stress effects that could interfere with the action of the agents. Accordingly, all treatments were given to the animals between 3:00 and 4:00 p.m. for 14 consecutive days.

### 4.6. Body Weight Changes Evaluation

Body weight was recorded daily throughout the experimental period using a calibrated digital scale to monitor general health status and detect any stress- or treatment-induced changes. Each rat was weighed at the same time each morning to minimize circadian variability (8:00 to 9:00 p.m.). The percentage change in body weight was determined based on the starting values of the experiment. Any significant alterations in body weight were recorded as indicators of stress severity or therapeutic efficacy [70].

### 4.7. Anxiety-like Behavioral Change Evaluation

Following the 14-day stress induction and treatment period, behavioral assessments were conducted to evaluate anxiety-like behaviors. On experimental days 15 and 16, the light/dark box and the elevated-plus maze (EPM) tests were performed in the morning from 9:00 to 12:00 p.m.

#### 4.7.1. Light/Dark Test

On day 15, the light/dark box test, following a previously established protocol, was conducted to assess anxiety-like behavior [71]. The apparatus consisted of two adjoining plastic compartments: a brightly lit compartment (30 × 30 × 32 cm, 250 lux) and a dimly lit compartment (30 × 32 × 32 cm, 50 lux), connected by an opening allowing free movement. Rats were individually placed in the light compartment, and behavior was monitored for 5 min via an overhead video camera. Recorded parameters included duration in each compartment and the frequency of transitions from dark to light. Lower transition frequency and reduced time in the light compartment indicated higher anxiety-like behavior [72].

#### 4.7.2. Elevated-Plus Maze (EPM) Test

The EPM was constructed from black plastic and consisted of an open-topped platform elevated 50 cm above the floor, featuring two open arms (50 × 10 cm) perpendicular to two closed arms (50 × 10 × 40 cm). Each rat was placed in the central square and allowed to explore for 5 min. Behavior was recorded using an infrared camera, measuring time spent in the open arms and the number of open arm entries. The anxiety index was calculated by averaging the proportion of time spent and entries in the open arms, subtracting from one, and dividing by two. Decreased duration and frequency of open-arm exploration, and a higher anxiety index, indicate increased anxiety-like behavior [73,74].

### 4.8. Tissue Collection and Sample Preparation

Twenty-four hours after the behavioral tests, deep euthanasia was performed by administering an overdose of 5% isoflurane via inhalation. Blood samples were collected through cardiac puncture, and key target tissues—including the brain, colon, and tibia—were harvested for subsequent analyses. Collected serum was used to measure brain-derived neurotrophic factor (BDNF), short-chain fatty acids (SCFAs), bile acids (BAs), polyamines, and osteocalcin using enzyme-linked immunosorbent assay (ELISA) kits. Whole brains and colons were rapidly excised, snap-frozen in liquid nitrogen, and kept at −80 °C for subsequent analyses for later assessment of protein expression related to neuroplasticity (synapsin-1 and PSD-95), barrier integrity (ZO-1 and occludin), and neuroinflammation (Iba1/AIF-1, a microglial/macrophage marker). The right tibia was dissected, and tissue adhesions were carefully removed before fixative incubation and decalcification. For histological examination, rats were transcardially perfused with cold phosphate-buffered saline (PBS, pH 7.4) and subsequently fixed in 4% paraformaldehyde (PFA) for 24 h. Fixed tissues, including brain, colon, and tibia, were embedded in paraffin, sectioned, and stained with hematoxylin and eosin (H&E) to examine tissue histomorphology.

### 4.9. Serum Biochemical Markers Evaluation

Blood was obtained via cardiac puncture between 9:00 a.m. and 12:00 p.m., after which serum was prepared. Serum levels of BDNF (Catalog No. E-EL-R1235, Elabscience, Houston, TX, USA), SCFAs (Catalog No. SL1669Ra, Sunlong Biotech, Hangzhou, China), BAs (Catalog No. 80461, Crystal Chem, Elk Grove Village, IL, USA), polyamines (Catalog No. ab239728, Abcam, Cambridge, UK), and osteocalcin (Catalog No. AC-12F1, Newcastle, UK) were measured using commercial ELISA kits and analyzed with a microplate reader.

### 4.10. Intestinal Permeability Evaluation

To evaluate intestinal barrier integrity, a FITC-dextran permeability assay was performed. Twenty-four hours after the behavioral tests, all rats from each experimental group were fasted for 6 h prior to the procedure, with free access to water. After the fasting period, rats received FITC-dextran (4 kDa, Sigma-Aldrich, St. Louis, MO, USA, 40 mg/mL) via oral gavage at 10 µL/g body weight in sterile PBS, pH 7.4 [75,76]. Exactly one hour after FITC-dextran administration, blood samples were collected via cardiac puncture under isoflurane anesthesia. After collection, blood samples were allowed to clot at room temperature for 30 min and then centrifuged at 3000× *g* for 15 min at 4 °C to obtain serum. Serum FITC-dextran concentrations were measured using a fluorescence spectrophotometer (excitation 485 nm, emission 535 nm), and levels were calculated based on a standard curve generated from known FITC-dextran concentrations, which were expressed relative to the control group (%) and served as an indicator of intestinal permeability. Elevated serum FITC-dextran levels reflected increased intestinal barrier disruption [3,75].

### 4.11. Western Blot Analysis

Following euthanasia of rats (*n* = 4/group), fresh hippocampus and colon were collected and lysed for protein extraction. Each tissue (50 mg) was homogenized in 500 µL of RIPA buffer mixed with protease and phosphatase inhibitors (Abcam, Cambridge, UK), initially using a POLYTRON PT 3100 homogenizer for 10 cycles, then subjected to sonication three times using a Vibra-Cell VCX-600 Ultrasonic Processor (SONICS & MATERIALS, INC., Newtown, CT, USA) at 20 Amp with 20% on/off cycles for 5 s each. The lysates were vortexed in three intervals of 10 min and centrifuged at 12,000 rpm for 20 min at 4 °C using a Thermo Scientific Fresco 21 microcentrifuge. Collected supernatants were analyzed for protein concentration using the Thermo Scientific Pierce BCA Protein Assay Kit. Twenty micrograms of protein per sample were separated on SDS-PAGE gels and subsequently transferred onto nitrocellulose membranes (Merck Millipore Ltd., Co. Cork, Ireland) following standard protocols. Membranes were blocked and incubated overnight at 4 °C with primary antibodies targeting ZO-1 (rat monoclonal antibody [mAb], 1:1000; catalog no. sc-33725), occludin (mouse mAb, 1:500; catalog no. sc-271842), and β-actin (mouse mAb, 1:1000; catalog no. sc-47778) from Santa Cruz Biotechnology (Dallas, TX, USA), as well as PSD-95 (rabbit mAb, 1:1000; catalog no. 3409S), synapsin-1 (rabbit mAb, 1:1000; catalog no. 5297S), and Iba1/AIF-1 (rabbit mAb, 1:1000; catalog no. 17198S) from Cell Signaling Technology (Trask Lane Danvers, MA, USA). Membranes were washed and then incubated with secondary antibodies linked to horseradish peroxidase (HRP) for 1 h at room temperature. These included HRP-conjugated anti-rat, anti-rabbit, and anti-mouse antibodies used at 1:5000. Signal detection was performed using the Immobilon Crescendo Western HRP Substrate (Merck-Millipore, Burlington, MA, USA), and images were acquired using the Azure Biosystems Western Blot Imaging System. Densitometry of protein bands was performed with ImageJ software (version 1.53, NIH Image, Bethesda, MD, USA). Protein levels were determined based on optical density measurements and normalized to β-actin to ensure accurate comparison across samples followed the method from Lapmanee et al. [3,38].

### 4.12. Histological Evaluations of the Hippocampus, Colon, and Tibia

The euthanized rats (*n* = 4/group) were carefully dissected; the hippocampus, colon and tibia and were fixed in 4% PFA for 24 h. Tissues were dehydrated in ethanol, cleared with xylene, embedded in paraffin, sectioned at 5 µm using a rotary microtome (Leica, Nussloch, Germany) and mounted onto coated glass slides. Brain tissues were sectioned to examine the hippocampal regions, focusing on the pyramidal cell layer thickness in cornu ammonis (CA) 1 and CA3, and the granule cell layer in the dentate gyrus. In addition, pyknotic cells—characterized by condensed nuclei—were counted in all subregions as indicators of neuronal injury [3]. Furthermore, for hippocampal assessments, every 6th section (a total of 10 sections per brain) was selected. Ten images per region were captured from both the left and right hemispheres of each rat to ensure comprehensive representation [77]. A 1 cm colonic segment was collected and assessed for mucosal architecture, goblet cell integrity, and inflammatory cell infiltration using a standardized histopathological scoring system [78]. Inflammation severity, epithelial damage, and goblet cell depletion were quantified using a scoring scale from 0 (no pathology) to 4 (severe pathology), as followed by Ding et al. [79]. Lastly, right tibial bones were decalcified in solution (Leica Biosystems, Germany) for 21 days [80] before being embedded in paraffin, sectioned, and examined for trabecular structure, osteoblast and osteoclast distribution, and percentage of trabecular area [81,82]. Tissue morphology was assessed by H&E staining and light microscopy (Olympus BX53, Olympus, Tokyo, Japan). Ten images were captured per section to ensure adequate sampling for both quantitative and qualitative analyses. All image analyses were performed independently by two blinded pathologists to minimize observer bias.

### 4.13. Statistical Analysis

Results are expressed as mean ± standard error of the mean (SEM). Normal distribution of data was evaluated with the Kolmogorov–Smirnov test. Two-group comparisons were performed using an unpaired Student’s *t*-test, while experiments involving more than two groups were analyzed by one-way ANOVA followed by Dunnett’s multiple comparisons test to enhance statistical power for control or vehicle comparisons. Results were considered statistically significant at *p* < 0.05. GraphPad Prism 10 (GraphPad Software, San Diego, CA, USA) was used for all statistical analyses and figure generation.

## 5. Conclusions

**Figure 8:**
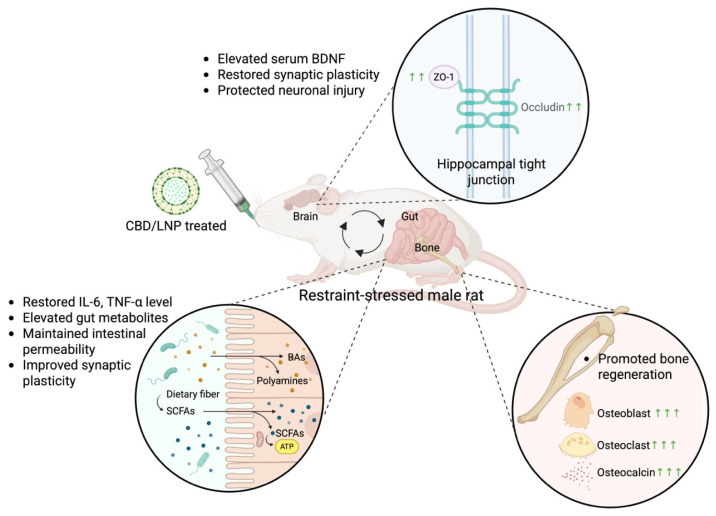
Summary of the present findings. As opposed to the untreated stressed group, CBD/LNP treatment in restraint-stressed male rats yielded the most effective therapeutic outcomes, including restoration of systemic inflammation (IL-6 and TNF-α levels), improvement in intestinal integrity, and elevation of gut metabolites (SCFAs, BAs, and polyamines). Synaptic plasticity markers were enhanced in both hippocampal and colonic regions. Additionally, the encapsulated form of CBD promoted bone regeneration by restoring osteoclast, osteoblast, and osteocalcin levels, and by inducing BDNF, PSD-95, and synapsin-1 expression for neuronal protection. This figure was created in BioRender. Thanawuth, K. (2025) https://BioRender.com/w0ameir (accessed on 1 September 2025).

These findings comprehensively indicate that chronic restraint stress in rodents impairs the gut–brain–bone axis through neuroinflammation, dysregulation of gut metabolites and intermodulatory markers, and structural deterioration. Repeated stress elevated systemic cytokines, resulting in reduced hippocampal and intestinal tight junctions, impaired BDNF expression, and disrupted bone remodeling. This pathophysiology was closely linked, suggesting that systemic inflammation and barrier dysfunction are key drivers of neurobehavioral and skeletal impairments. Among the interventions tested, CBD/LNPs emerged as the most effective therapy, outperforming both traditional antidepressants (i.e., ATX and ECS) and native CBD in restoring barrier integrity, synaptic markers, and bone microstructure. The superior outcomes are attributed to the enhanced bioavailability and tissue-targeting properties of lipid nanoparticles, facilitating multi-organ delivery under inflammatory conditions (Figure 8). This work provides new evidence supporting the use of nanoformulated CBD as a precision-driven therapeutic platform for complex, stress-induced comorbidities. Furthermore, these findings have broader relevance to the Sustainable Development Goals (SDGs), with emphasis on SDG 3, Good Health and Well-being, by highlighting strategies to prevent and treat chronic stress-related disorders. It also underscores the importance of targeting systemic pathways—rather than isolated symptoms—in improving resilience and recovery across multiple physiological domains. Further translational research into CBD-based nanomedicines may yield novel strategies for enhancing multi-organ health and well-being.

## Data Availability

The data presented in this study are available on request from the corresponding author.

## Abbreviations

The following abbreviations are used in this manuscript:

AIF-1	Allograft inflammatory factor 1
ATX	Atomoxetine
BA	Bile acid
BDNF	Brain-derived neurotrophic factor
CA	Cornu ammonis
CBD	Cannabidiol
CBD/LNP	Cannabidiol-loaded lipid nanoparticles
CON	Control
DG	Dentate gyrus
ECS	Escitalopram
ELISA	Enzyme-linked immunosorbent assay
FICT	Fluorescein isothiocyanate
GCL	Granule cell layer
H&E	Hematoxylin and eosin
HPA	Hypothalamic–Pituitary–Adrenal axis
HRP	Horseradish peroxidase
Iba1	Ionized calcium-binding adapter molecule 1
IL	Interleukin
NRI	Norepinephrine reuptake inhibitor
PBS	Phosphate-buffered saline
PFA	Paraformaldehyde
PCL	Pyramidal cell layer
PSD-95	Postsynaptic density protein 95
SCFA	Short-chain fatty acid
SEM	Standard error of the mean
SSRI	Selective serotonin reuptake inhibitor
SYN	Synapsin-1
TNF-α	Tumor necrosis factor-alpha
VEH	Vehicle
ZO-1	Zonula occludens-1
